# Using Anatomy-Based Fitting to Reduce Frequency-to-Place Mismatch in Experienced Bilateral Cochlear Implant Users: A Promising Concept

**DOI:** 10.3390/jpm13071109

**Published:** 2023-07-08

**Authors:** Anja Kurz, David Herrmann, Rudolf Hagen, Kristen Rak

**Affiliations:** Department of Otorhinolaryngology, Head & Neck Surgery, Comprehensive Hearing Center, University of Würzburg, Josef-Schneider-Str. 11, 97080 Würzburg, Germany

**Keywords:** cochlear implant, anatomy-based fitting, frequency-to-place mismatch, speech perception

## Abstract

Fitting cochlear implant (CI) users can be challenging. Anatomy-based fitting (ABF) maps may have the potential to lead to better objective and subjective outcomes than conventional clinically based fitting (CBF) methods. ABF maps were created via information derived from exact electrode contact positions, which were determined via post-operative high-resolution flat panel volume computer tomography and clinical fitting software. The outcome measures were speech understanding in quiet and noise and self-perceived sound quality with the CBF map and with the ABF map. Participants were 10 experienced bilateral CI users. The ABF map provided better speech understanding in quiet and noisy environments compared to the CBF map. Additionally, two approaches of reducing the frequency-to-place mismatch revealed that participants are more likely to accept the ABF map if their electrode array is inserted deep enough to stimulate the apical region of their cochlea. This suggests an Angular Insertion Depth of the most apical contact of around 720°–620°. Participants had better speech understanding in quiet and noise with the ABF map. The maps’ self-perceived sound quality was similar. ABF mapping may be an effective tool for compensating the frequency-to-place mismatch in experienced bilateral CI users.

## 1. Introduction

Cochlear implant (CI) provision is the standard treatment option in many countries for people with unilateral or bilateral severe-to-profound sensorineural hearing loss. In bilateral implantation, there remains some debate regarding if it is better to perform simultaneously, i.e., both CIs in one surgical event, or sequentially, i.e., separate surgeries performed weeks to years apart [1,2,3]. An important factor in determining who receives a CI (or two CIs) is funding priorities where the candidate lives [4,5]. In some countries, simultaneous bilateral CI provision in children younger than three years of age with bilateral sensorineural hearing loss is given funding priority over CI provision for other age groups and types of hearing loss [6]. In other countries, only unilateral implantation is funded in people with bilateral deafness [6,7,8]. A study on people who received bilateral CIs in their childhood demonstrated significant benefits of CI use, not only in the time frame where hearing and speech intelligibility develop, but also up to adulthood [9]. In adults, hearing loss is often progressive and asymmetrical so one ear may reach the indication for a CI earlier than the contralateral side [4,5]. In these cases, a CI is usually chosen for the poorer hearing side while the other ear may still benefit from acoustic amplification (e.g., via a conventional hearing aid). In clinical practice, we often encounter adult bilateral CI users with postlingual deafness who received their CIs sequentially and favor their first or second implanted ear, like being left- or right-handed. To determine the underlying causes for this, studies have investigated factors that would predict the outcome of the second side [10,11,12]. A recent systematic review on performance with a second CI in sequential implantations showed that age at implantation, duration of hearing loss, and the length of the interval between implant surgeries did not predict postoperative speech perception outcomes [12]. A recent paper suggests that first-side CI scores on the AzBio Sentence Test in quiet may predict long term (≥12 months) second-side CI performance [11]. Another research group measured the squelch effect to determine if there would be a first ear advantage in sequential implantations. In their study, some subjects could take advantage of the squelch effect, but the majority could not [13].

As described by Pieper et al. [14] there may be factors such as sound processing latency along the auditory pathway (evoked by an audio processor or hearing aid), tonotopic misaligned amplified frequencies, and loudness (level) differences when fitting CI recipients, especially bimodal users (i.e., those with a CI in one ear and a hearing aid in the contralateral ear). Their paper further suggests experimental methods to reduce the mismatch. However, a clinically applicable approach for bilateral CI users is so far missing. To this end, cochlear imaging may be of benefit. Indeed, recently, there has been increasing interest in determining anatomical structures of the cochlea and defining frequency areas more precisely [15,16,17]. New imaging surgical planning software can display the exact position of the electrode array after surgery. This information is especially useful when fitting the audio processor because knowledge of the exact positions of electrode contacts within the cochlea (i.e., insertion depth) has the potential to reduce frequency-to-place mismatch, which is the mismatch between the tonotopy of the electrode array relative to tonotopy of the cochlea, and thereby improve CI users’ speech understanding [18,19].

The new concept called “anatomy-based fitting (ABF)” has the potential to systematically decrease the frequency-to-place mismatch by providing tonotopic electric stimulation using the CI user’s accustomed audio processor. ABF is a concept pioneered by MED-EL (MAESTRO 9.0 System Software|MED-EL Pro (medel.pro)), on which there are few publications [20]. In bilateral CI users, applying ABF on both sides additionally reduces the tonotopic asymmetry between the ears. Since utilizing the full potential of binaural hearing requires matched inputs of the binaural neurons in the brainstem, it was hypothesized that using an ABF map would also improve spatial release from masking. The first published results on ABF mapping are promising, but contain data on only 3 CI users [20]. It was therefore necessary to conduct a study on a larger cohort of CI users. The aim of the present investigation was to determine if using an ABF-generated fitting map improves speech perception in quiet and in noise, self-perceived sound quality and measures of binaural processing (squelch and spatial release from masking) in adult bilateral CI users who were sequentially implanted. 

## 2. Materials and Methods

### 2.1. Participants

To participate in the study, candidates had to meet all of the following inclusion criteria: (1) have an available post-operative flat panel volume computed tomography (fpVCT) image with a secondary reconstruction of 99 µm taken after their CI surgery (taken after the second implantation), (2) be at least 18 years old at the study start, (3) have postlingual bilateral deafness, (4) be a bilateral CI user with at least 6 months of experience with the SONNET 2 or RONDO 3 audio processor (MED-EL, Innsbruck, Austria), (5) have at least ten active electrode contacts on each side, (6) use the FSP, FS4, or FS4-p sound-coding strategy on each side, (7) have a speech understanding with the individual CI of ≥25% on a monosyllables test at 65 dB sound pressure level (SPL) or a speech reception threshold (SRT) of ≤20 dB signal-to-noise-ratio (SNR) on a sentence-in-noise test, (8) be willing and able to give feedback on the fitting map and process, and (9) give their signed and dated informed consent before participating in any study-related procedures. Candidates that did not fulfill the inclusion criteria were excluded. 

### 2.2. ABF Procedure

The post-operative position of individual electrode contacts in both cochleae was identified in fpVCT using clinical surgical planning software (OTOPLAN^®^, software version 3, CASCINATION AG, Bern, Switzerland). This information was then imported into clinical fitting software MAESTRO 9.0.5 (MED-EL, Innsbruck, Austria) and displayed as individual electrode contacts within the manufacturer’s pre-set frequency band distribution (range 70–8500 Hz) of the audio processors. 

An analysis of the Angular Insertion Depth (AID) for contact E1 revealed an uneven coverage of the apical region in all 10 participants (20 ears). Based on exploratory fittings in clinical routine as described above, we therefore defined two groups: Group 1 had a least one ear with the first electrode contact E1 above 620° and a shorter electrode array on the contralateral side, not reaching 620°. Group 2 had a shallower insertion with E1 below 570°. 

For Group 1, a bilateral ABF fitting was created by adjusting filter bands in MAESTRO on each side to the respective tonotopic electrode frequencies, so that sides were matched by matching each side individually to electrode frequencies calculated from Greenwood [21] in OTOPLAN. In detail, the following procedure was applied: firstly, the reference ear (i.e., the ear with better hearing) was determined via subjective feedback. Secondly, the Organ of Corti (OC) pre-settings were used within the reference ear as per the manufacturer’s implementation of ABF mapping. To reduce any unfavorable sounds, the Most Comfortable Levels (MCL) were adapted, and it was confirmed that the center frequency for the most apical electrode contact (E1) was within 100–200 Hz. 

**Figure 1:**
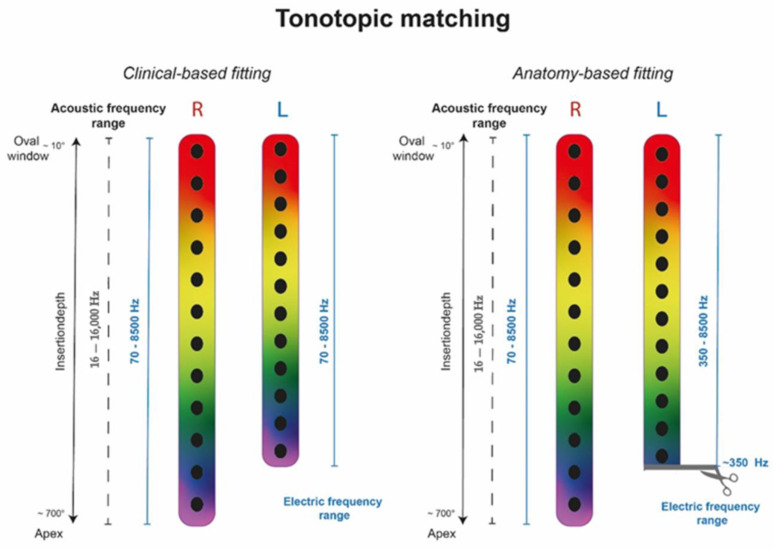
Scheme illustrating tonotopic matching in the standardized versus the anatomy-based fitting. The colorful cochleae represent the acoustic frequency distribution ranging from 16–16,000 Hz and the electric frequencies (in blue) distribution from 70–8500 Hz in the audio processor for the right (R) and left (L) side. The scissors indicates the shift of the frequency cut-off up to 350 Hz in the anatomy-based fitting example.

In the next step, the electrode contacts were displayed in the fitting software for the contralateral ear. In participants implanted with a standard-length array but with shallower insertion (400°–500° AID), the OC frequency of the apical electrode contact lay outside the first frequency band (to be visualized in the fitting software). To cover the OC frequency of the apical electrode, the lower frequency limit of the filter bank was shifted upwards accordingly. Consequently, lower frequencies were cut off to allow tonotopic matching (as displayed in Figure 1). For example, participant #1 had a standard-length array on the right side (AID of E1: 730°) and a shorter length array on the left side (AID of E1: 510°). In this case, the right side was the reference side and ABF was activated as implemented in the fitting software. On the left side, E1 was at 510°, meaning the OC frequency was around 400 Hz. To allow tonotopic matching, the lower frequency limit of the filter bank was moved up to 350 Hz, assuming matched tonotopicity.

For Group 2, a bilateral ABF fitting was created by calculating the ratio between tonotopic electrode frequencies (as determined in OTOPLAN) and filter band frequencies on one side and using the same ratio for calculating filter band frequencies from tonotopic electrode frequencies on the other side. In other words, filter bands on both sides were equally transposed in reference to the respective tonotopic electrode frequencies. Thus, here, both sides have not been matched to Greenwood in absolute terms, but both sides are equally transposed to Greenwood and are thus matched. This principle was developed based on a pilot investigation for patients that did not accept the tonotopic fitting as suggested for Group 1. The developed formula was the first step in incorporating the findings from the OTOPLAN analysis into the fitting. However, a patient-specific adjustment of the frequency bands is still necessary. The formula can also be extended, e.g., by calculating the distance to the neighboring electrode. In this “transposition” approach, the reference ear (i.e., the ear with better hearing) was determined via subject feedback, and MCL levels were confirmed for comfortable loudness. The reference ear remained with the clinically based fitting (CBF) map, applying the center filter frequencies as provided per default in the MAESTRO fitting software. In the contralateral ear, the frequency bands were determined using the following calculation:

$$c_{contralateral,e}=\frac{foc_{contralateral,e}}{foc_{reference,e}}\cdot fc_{reference,e}$$

For example: participant #8 had a FLEX28 on the right side (AID of E1: 518.7°) and a FLEX28 array on the left side (AID of E1: 557.8°); the left side is the reference ear. The recalculation of the center frequencies by applying the mentioned formula results in (see Table 1 below):

### 2.3. Assessment

All assessments were conducted in a sound-isolated room using active loudspeakers (M52 Klein and Hummel, Georg Neumann GmbH, Berlin, Germany) and a pre-amplification system (Focusrite Scarlett 18i202nd Generation, High Wycombe, UK). A custom program using the software Matlab (Math Works, Natrick, MA, USA) was used to conduct speech understanding tests. The participants were seated in the center of a semi-circle of nine loudspeakers equally spaced in the frontal horizontal plane, with a radius of 1.5 m. Sound was played from only the speakers on the far left, center, and far right (#1, 5, and 9).

Participants used their own audio processers during testing. Testing took place in two intervals: baseline and three months post-baseline. During baseline testing, participants used their accustomed CBF map. Participants were then re-fitted using ABF and asked to use only their ABF map for three months. At three months post-baseline, tests were repeated using the same measures.

#### 2.3.1. Speech Understanding in Quiet

Speech understanding in quiet was assessed using the German Freiburg Monosyllables test at 65 dB SPL [22] with the speech signal coming from the front (S0). Speech in quiet was assessed in three setups: unilaterally with only the reference side CI (hereafter, simply “reference”), unilaterally with only the contralateral side CI (hereafter, simply “contralateral”), and bilaterally. Scores are reported as percentage correct.

#### 2.3.2. Speech Understanding in Noise

Speech understanding in noise was assessed using the Oldenburg MATRIX test with a variable marker and a constant speech signal (at 65 dB SPL) [23]. The Oldenburg MATRIX test uses an adaptive test procedure to estimate the signal-to-noise (SNR) ratio required for 50% correct understanding of words. The order of the presentations and the test lists were randomized to minimize the effects of training and fatigue. To quantify the binaural hearing effects such as squelch and spatial release from masking (SRM), the Oldenburg MATRIX test was conducted in two spatial configurations: S0N0 (speech and noise from the front; 0° azimuth) and S0Ncontral (speech from the front, 0° azimuth; noise directed at the contralateral ear, ±90° azimuth). 

#### 2.3.3. Binaural Effects

Binaural effects were calculated following Schleich et al. [24]. 

The squelch effect describes the benefit resulting from spatial separation between the signal source and the noise source and is calculated in our setting as follows:

Squelch (dB) = SRTRef only (S0Ncontral) − SRT (S0Ncontral)

The summation effect is the advantage of hearing with cochlear implants with identical signals arriving at both sides in the S0N0 condition.

Summation (dB) = SRTRef only (S0N0) − SRTBilateral (S0N0Sum)

Spatial release from masking is referred to the improvement in speech intelligibility scores when speech and noise are spatially separated, and is calculated as follows:

SRM (dB) = SRTBilateral (S0N0) − SRTBilateral (S0Ncontral)

#### 2.3.4. Self-Perceived Sound Quality

Participants’ self-assessed their sound quality using the Hearing Implant Sound Quality Index (HISQUI19) [25]. The HISQUI19 consists of 19 questions answerable on a scale from “Always”, which is worth 7 points, to “Never”, which is worth 1 point. “Not applicable” is also an answer option, and is worth 0 points. The total score is obtained by adding the numerical values of all 19 questions. Results are qualified as follows: <30 indicates “very poor sound quality”, 30–60 points indicates “poor sound quality”, 61–90 points indicates “moderate sound quality”, 91–110 points indicates “good sound quality”, and >111 points indicates “very good sound quality”.

### 2.4. Statistical Analysis Methods

The mean and the standard deviation (±SD) were used to report participants’ characteristics (e.g., age at testing, CI hearing experience with each ear), and to describe the study outcomes. Results of the Kolmogorov–Smirnov test and the Shapiro–Wilk test confirmed approximately normal distribution of the study outcomes. Paired samples *t*-tests were applied to examine if a significant difference between the CBF map and the ABF map exists for each test condition (i.e., reference ear, contralateral ear, bilateral). Univariate General Linear Models with “map difference” (∆ABFmap—CBFmap) as a dependent variable were used to look for a significant influence of the variable “group” (Group 1 vs. Group 2) as a fixed factor for each test condition. The significance level was set to *p* ≤ 0.05. The problem of multiplicity resulting of multiple comparisons was solved by adjusting the *p*-values using the Holm–Bonferroni method per test outcome. 

IBM SPSS Statistics Version 25 (IBM, Armonk, New York, NY, USA) was used for the analyses. The generation of figures was performed using Prism GraphPad (Version 8.1, GraphPad Software, San Diego, CA, USA).

## 3. Results

### 3.1. Participants

Ten experienced bilateral CI users were included in the study. Participants were a mean 54.1 years old at time of hearing loss (SD: 17.6 years) and had a mean hearing experience with a CI of 7.6 years (SD: 4.9 years) for the first-implanted ear and 1.6 years (SD: 0.9 years) for the second-implanted ear. All participants had postlingual hearing loss. Group 1 consisted of participants 1, 2, 3, 6, 9, and 10. Group 2 consisted of participants 4, 5, 7, and 8. Participant #7 was included in Group 2 because they had a deep insertion of a FLEX24 array (AID 639.9°) which caused an unsatisfactory outcome. Using a map based on tonotopic fitting had also yielded unsatisfactory hearing results in this participant. 

Participants’ demographics are presented in Table 2.

### 3.2. Speech Understanding in Quiet

**Figure 2:**
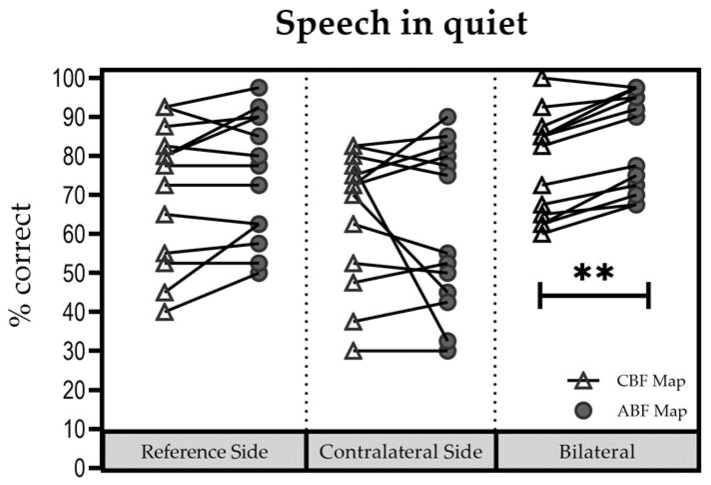
Speech understanding in quiet; scores for each participant in each setup. ** Statistically significant difference (*p* < 0.01).

Figure 2 shows individual results for all three listening conditions. Notably, in the bilateral conditions, subjects showed improvements with ABF. Mean (±SD) scores were as follows: for the reference side, 65% (±15.81%) with the CBF map [Group 1: 70% (±16.1); Group 2: 57.5% (±13.9%)] and 69.75% (±15.07%) with the ABF map [Group 1: 74.6% (±16.4%); Group 2: 62.5% (±10.8%)]; for the contralateral side, 60.25% (±17.54%) with the CBF map [Group 1: 65% (±18.3%); Group 2: 53.1% (±15.8)] and 60.5% (±20.34%) with the ABF map [Group 1: 62.9% (±23.3%); Group 2: 56.8% (±17.6)], and for the bilateral listening condition, 72.75% (±10.57%) with the CBF map [Group 1: 76.7% (±9.4%); Group 2: 66.9% (±10.5%)] and 80.5% (±11.95%) with the ABF map [Group 1: 83.7% (±12.8%); Group 2: 75.6% (±10.1%)].

No significant influence of “group” (Group 1 vs. Group 2) on “maps” (∆ABFmap—CBFmap) was found for each test condition (reference ear: (F(1; 8) = 0.007; *p* = 0.934); contralateral ear (F(1; 8) = 0.595; *p* = 0.463); bilateral (F(1; 8) = 0.617; *p* = 0.455). Therefore, results were pooled for further analyses. No significant difference between the CBF map and the ABF map was found for the reference ear (t = −2.111; df = 9; *p* = 0.064), and for the contralateral ear (t = −0.069; df = 9; *p* = 0.946). In the bilateral setup the difference between the two maps was significant in favor of the ABF map (t = −7.619; df = 9; *p* < 0.001).

### 3.3. Speech Understanding in Noise

#### 3.3.1. S0N_contral_ Configuration

**Figure 3:**
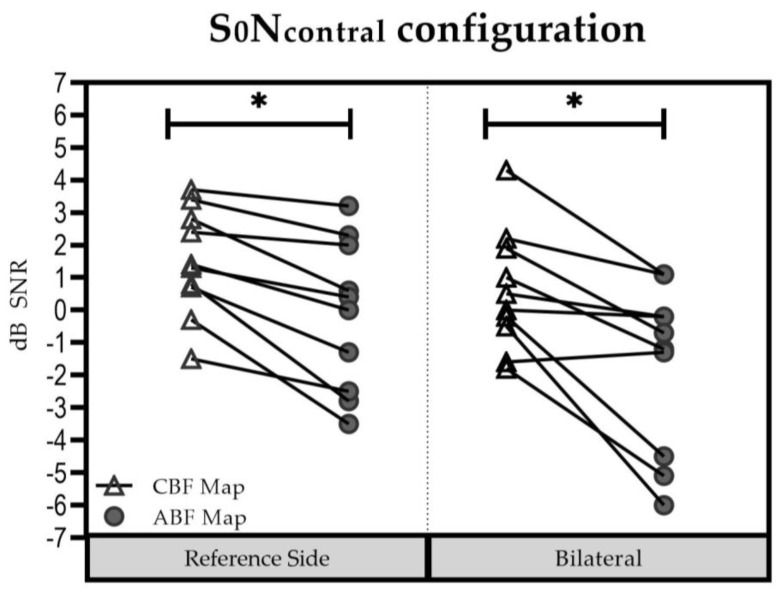
Speech understanding in the S0Ncontral configuration for each participant in each setup. Stars mark statistically significant differences between the CBF and ABF Map (* *p* < 0.05).

In the reference side (Figure 3), the mean (±SD) was 1.5 dB SNR (±1.65 dB SNR) with the CBF map [Group 1: 0.90 dB SNR (±1.63 dB SNR); Group 2: 2.33 dB SNR (±1.44 dB SNR)] and −0.11 dB SNR (±2.36 dB SNR) with the ABF map [Group 1: −0.63 dB SNR (±2.22 dB SNR); Group 2: 0.67 dB SNR (±2.68 dB SNR)]. The difference was significant in favor of the ABF map (t = 4.271; df = 9; *p* = 0.002).

In the bilateral listening condition, the mean (±SD) was 0.58 dB SNR (±1.85 dB SNR) with the CBF map [Group 1: −0.03 dB SNR (±1.46 dB SNR); Group 2: 1.50 dB SNR (±2.20 dB SNR)], and −1.70 dB SNR (±2.57 dB SNR) with the ABF map [Group 1: −2.17 dB SNR (±2.09 dB SNR); Group 2: −1.00 dB SNR (±3.39 dB SNR)]. The difference was significant in favor of the ABF map (t = 3.865; df = 9; *p* = 0.004). 

No significant influence of “group” (Group 1 vs. Group 2) on “maps” (∆ABFmap—CBFmap) was found for the reference ear: F(1; 8) = 0.021; *p* = 0.888) and the bilateral test condition (F(1; 8) = 0.083; *p* = 0.780. Therefore, results were pooled for further analyses. Results for the reference side were significant in favor of the ABF map (t = 4.271; df = 9; *p* = 0.002). Likewise, results for the bilateral setup were also significant in favor of the ABF map (t = 3.865; df = 9; *p* = 0.004).

#### 3.3.2. S0N0 Condition

**Figure 4:**
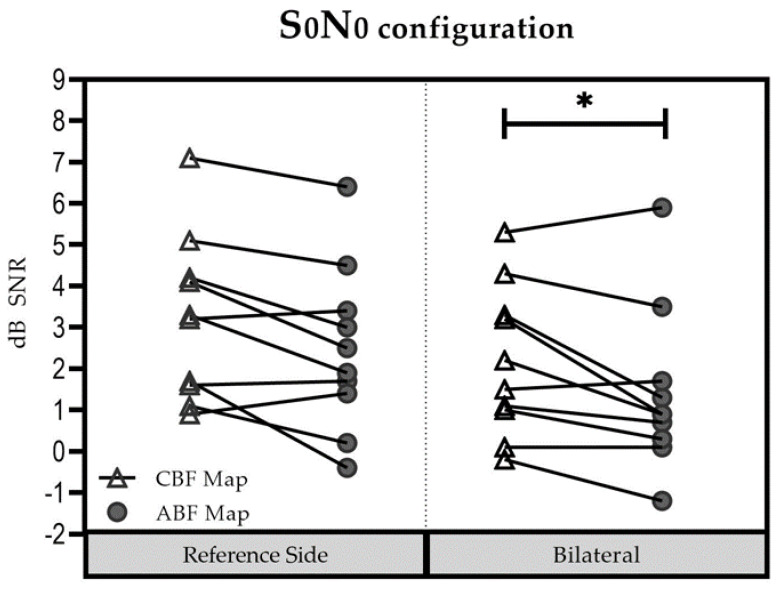
Speech understanding in the S0N0 configuration for each participant in each setup. * Statistically significant difference (*p* < 0.05).

In the reference side (Figure 4), the mean (±SD) was 3.23 dB SNR (±1.97 dB SNR) for the CBF map [Group 1: 2.53 dB SNR (±1.63 dB SNR); Group 2: 4.27 dB SNR (±2.21 dB SNR)] and 2.58 dB SNR (±2.06 dB SNR) for the ABF map [Group 1: 2.07 dB SNR (±1.91 dB SNR); Group 2: 3.35 dB SNR (±2.32 dB SNR)]. The difference between the two maps was not significant (t = 0.713; df = 9; *p* = 0.494).

In the bilateral listening condition, the mean (±SD) was 2.18 dB SNR dB (±1.81 dB SNR) for the CBF map [Group 1: 1.87 dB SNR (±1.77 dB SNR); Group 2: 2.65 dB SNR (±2.04 dB SNR)], and 1.41 dB SNR (±1.98 dB SNR) for the ABF map [Group 1: 1.05 dB SNR (±1.58 dB SNR); Group 2: 1.95 dB SNR (±2.65 dB SNR)]. Performance was better with the ABF map compared to the CBF map, but did not reach significance (t = 2.626; df = 9; *p* = 0.028). 

No significant influence of “group” (Group 1 vs. Group 2) on “maps” (∆ABFmap—CBFmap) was found for the reference ear: (F(1; 8) = 0.054; *p* = 0.822) and the bilateral test condition (F(1; 8) = 0.034; *p* = 0.858; summation effect (F(1; 8) = 0.335; *p* = 0.579; SRM (F(1; 8) = 0.697; *p* = 0.428). Results were thus pooled for further analysis. For the reference side, the difference between the two maps was not significant (t = 0.713; df = 9; *p* = 0.494). In contrast, for the bilateral setup, results showed significantly better performance with the ABF map compared to the CBF map (t = 2.626; df = 9; *p* = 0.028).

#### 3.3.3. Binaural Effects

**Figure 5:**
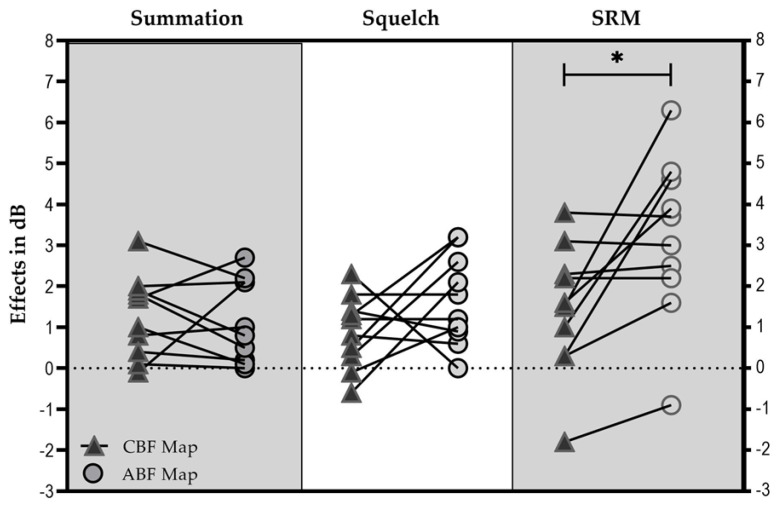
Individual results with each map for summation, squelch and SRM. * Statistically significant difference (*p* < 0.05).

The squelch effect (Figure 5) was on average 0.89 dB SNR (±0.89 dB SNR) with the CBF map [Group 1: 0.93 dB SNR (±0.93 dB SNR); Group 2: 0.83 dB SNR (±0.95 dB SNR)], and 1.66 dB SNR (±1.10 dB SNR) with the ABF map [Group 1: 1.53 dB SNR (±1.22 dB SNR); Group 2: 1.85 dB SNR (±1.03 dB SNR)].

The summation effect was on average 1.27 dB SNR (±1.00 dB SNR) with the CBF map [Group 1: 0.95 dB SNR (±0.88 dB SNR); Group 2: 1.75 dB SNR (±1.10 dB SNR)], and 1.17 dB SNR (±1.01 dB SNR) with the ABF map [Group 1: 1.02 dB SNR (±0.92 dB SNR); Group 2: 1.40 dB SNR (±1.24 dB SNR)].

SRM was on average 1.43 dB SNR (±1.59 dB SNR) with the CBF map [Group 1: 1.90 dB SNR (±1.44 dB SNR); Group 2: 0.73 dB SNR (±1.75 dB SNR)], and 3.17 dB SNR (±1.99 dB SNR) with the ABF map [Group 1: 3.22 dB SNR (±1.08 d SNR). Group 2: 3.1 dB SNR (±3.16 dB SNR)].

No significant influence of “group” (Group 1 vs. Group 2) on “maps” (∆ABFmap—CBFmap) was found for the bilateral effects (squelch effect (F(1; 8) = 0.145; *p* = 0.713; summation effect (F(1; 8) = 0.335; *p* = 0.579; SRM (F(1; 8) = 0.697; *p* = 0.428). Therefore, results were pooled for further analyses. No significant difference could be found between maps for the squelch (t = −1.481; df = 9; *p* = 0.173) and summation effect (t = 0.295; df = 9; *p* = 0.775). In contrast, statistical analysis showed a significant difference for SRM (t = −2.850, df = 9; *p* = 0.019).

### 3.4. Self-Perceived Sound Quality

**Figure 6:**
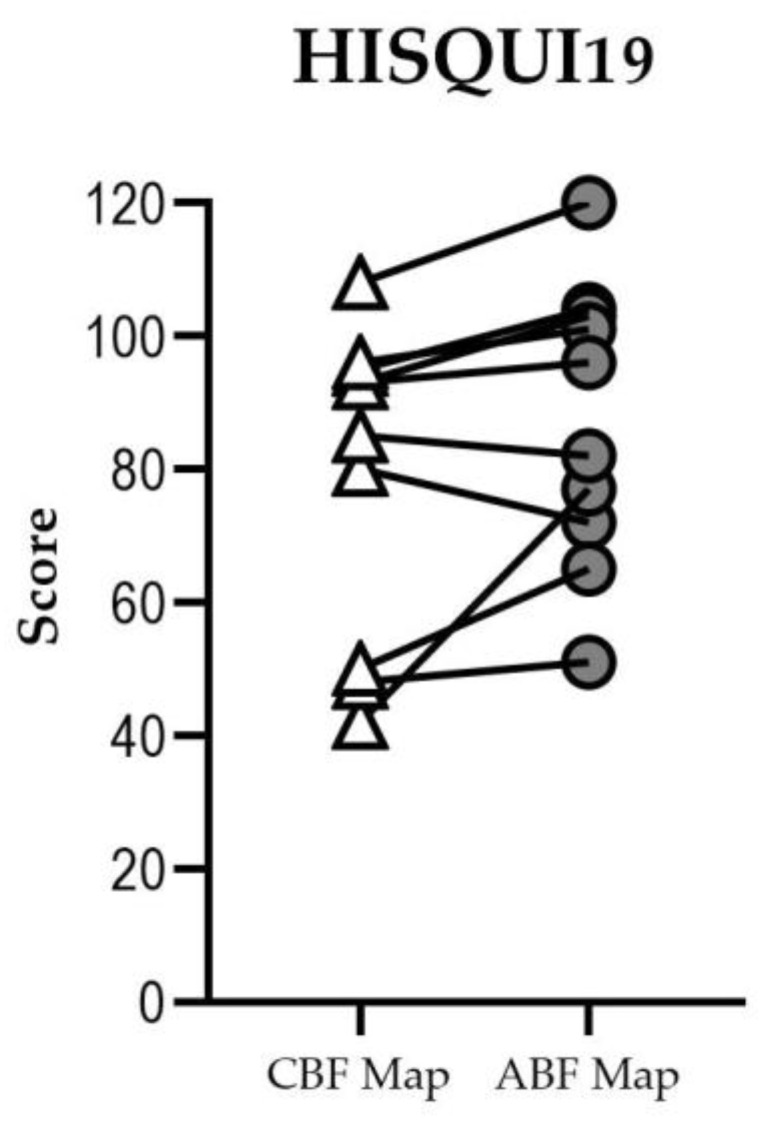
HISQUI19 scores for each individual when judging with the CBF map and the ABF map in the bilateral condition. Higher scores indicate better self-perceived sound quality.

Eight of ten participants had a higher HISQUI19 score with the ABF map than with the CBF map in the bilateral condition (Figure 6). Three participants improved their self-perceived sound quality: participant #3 from “poor” to “moderate”, subject #7 from “moderate” to “good”, and participant #10 from “good” to “very good”. Two participants had a lower score with the ABF map, but in neither case did their qualitative result change. 

With the CBF map, five participants perceived “good” sound quality, two “moderate”, and three “poor” sound quality. With the ABF map, one participant rated a “very good” sound quality, four “good”, four “moderate, and one a “poor” sound quality. Total mean score was 79.0 (SD: 22.3) for the CBF map and 87.1 (SD: 20.1) for the ABF map. The median for both fittings was 89, showing non-significant difference between the two groups. 

## 4. Discussion

This study aimed to determine if ABF maps would improve speech understanding and self-perceived sound quality, as compared to using the usual CBF map, in experienced bilateral adult CI users. These aims were accomplished: results showed that using the ABF map significantly improved speech understanding, both in quiet and in noise, and may lead to better self-perceived sound quality. Reducing the tonotopic mismatch between the ears also significantly improved binaural processing, as demonstrated by a significantly improved spatial release from masking. 

Because binaural processing can only be accomplished by matching the cues that arrive on both sides, we suggest that ABF is a suitable tool to provide matched inputs in bilateral CI recipients. As this is an ambitious goal, its application and acceptance in our study recipients was not self-evident at the beginning of this study. This was the motivation to develop two approaches which incorporated information about the exact electrode position in the individuals’ cochleae. Our analyses indicated that the AID of the first apical electrode contact determined which approach of ABF, either the tonotopic matching or the zipping principle, was accepted. 

ABF maps were immediately accepted by all participants whose first electrode contact was at an AID of between 730° and 620°, which corresponds to OC frequencies of 100–230 Hz (as displayed in the MAESTRO fitting software). These participants were therefore allocated to Group 1, and mostly had been implanted with a STANDARD electrode array (31.5 mm). In participants with shallower insertions, i.e., not reaching ~ 600° to 620°, tonotopic matching was not accepted which led to the development of the transposition principle. The transposition principle is inconsistent with tonotopicity at the first place when compared to Group 1, but consistent within the frequencies of the two ears. Both approaches use tonotopic information to improve the fitting, thereby justifying the term ABF.

Our results show that, with the ABF mapping, speech understanding in quiet increased slightly (but not significantly) in the reference ear-only setting and significantly in the bilateral set up. Further, average speech understanding in quiet in the contralateral ear-only mode slightly decreased with ABF, although not significantly. The reason for this might be a change in sound quality (for the worse) and poorer speech perception for that ear alone as a result of restricting the frequency in the second ear for ABF when compared to the frequency range used in CBF. However, speech understanding in quiet increased significantly in the bilateral setting, thereby demonstrating the benefit of minimizing frequency-to-place mismatch in bilateral CI users, even at the cost of performance with the contralateral ear alone.

To determine the effectiveness for speech perception in noise, we reported raw results but also calculated binaural effects. As shown in Figure 5, a non-significant summation effect was found between CBF and ABF mapping. At this point, it needs to be asked how large summation effects between fittings in bilateral CI users can be in general. Dorman et al. [26] compared binaural cues for adult bilateral CI users with bimodal users and CI users with high levels of residual hearing preservation and found a mean improvement of 1–2 dB for the different subject groups. In an older study by Schleich et al. [24], an average summation effect of 2 dB was found in bilateral CI users. In the present study, we did not expect to find significant improvements in the S0N0 condition between CBS and ABF mapping. In the paper by Pieper et al. [14], it was suggested that the dimensions of latency, loudness, and frequency-to-place alignment need to be aligned in order to provide interaural processing cues. In our study, we recruited experienced bilateral CI users anticipating that the dimensions of latency and loudness could be ignored (due to bilateral electrical processing and loudness matching prior to fitting). The purpose of our study was to provide a clinically applicable tool to reduce the frequency-to-place mismatch by integrating OTOPLAN data. Hence, the successful reduction of frequency-to-place mismatch showed significantly better results in calculating spatial release from masking, suggesting that binaural cues can be better provided using the ABF fitting approach.

A limitation of the present study is the limited amount of data. As more CI users will be fit with ABF in the future, more data will be available to support (or fail to support) our theory. The post-operative availability of high-resolution imaging allowed us to bring all factors such as insertion depth, cochleae size, and exact electrode contact position together to personalize and optimize the fittings. It has been shown before that in lower resolution imaging of about 400–600 µm, like normal computer tomography, cochlear size is underestimated, which results in an inaccurate measurement of the cochlear duct length and incorrect determination of the respective frequencies [27]. A practical limitation is that not every clinic has access to high-resolution imaging post-operatively and that cost coverage by health insurance or governments may prevent clinics from using this image-based adjustment. 

From a clinical perspective, we needed to plan 5 to 10 min more time to import the fpVCT post-surgery and apply the surgical planning software. The frequent use of the surgical planning software and the automated electrode detection algorithm, as implemented, reduced the preparation time substantially. When fitting, the shifting of frequency bands is visually easy and understandable and can be seamlessly implemented in clinical routine, especially when working with CI users who are unsatisfied with their hearing performance. 

## 5. Conclusions

ABF can be used to fit experienced bilateral cochlear implant users; it improves speech perception, especially in noise, and self-perceived sound quality. ABF fitting is a promising approach to match the input from both CIs, thereby providing access to binaural processing.

## Figures and Tables

**Table 1:** Applying the transposition principle. Fx default are center frequencies as used in clinical fittings in the MASTRO fitting software (SW); OC value contralateral ear and reference ear (in Hz) are available in the PowerPoint report provided by OTOPLAN after individual detection of the electrode contacts.

Electrode Contact	Fx Default (Maestro SW)	OC Frequency Contralateral Ear	OC Frequency Reference Ear	Recalculated “Transposed” Frequency
1	120	394	312	152
2	235	571	465	289
3	384	837	664	484
4	580	1141	932	710
5	836	1604	1308	1025
6	1175	2155	1732	1462
7	1624	2758	2406	1862
8	2222	3519	3121	2505
9	3020	4411	4013	3320
10	4084	5687	5213	4455
11	5507	7452	6773	6059
12	7410	10,551	9197	8501

**Table 2:** Demographic information for each participant.

Participants	AGE (Years)	Implanted Side	CI Experience (Years)	Implant	Electrode Array	E1 Angular Insertion Depth	Ref. Ear
1	77	Right	2	SYNCHRONY2	FLEX26	498.0°	Left
		Left	14	PULSAR	STANDARD	736.0°	
2	65	Right	3	SYNCHRONY2	FLEX28	457.7°	Right
		Left	1.5	SYNCHRONY2	FLEXSoft	671.6°	
3	57	Right	7.5	SONATA	STANDARD	790.0°	Right
		Left	2	SYNCHRONY2	FLEX26	519.0°	
4	61	Right	2.5	SYNCHRONY2	FLEX28	555.5°	Left
		Left	10.5	SYNCHRONY	FLEX28	529.5°	
5	57	Right	3	SYNCHRONY2	FLEX28	406.2°	Left
		Left	1	SYNCHRONY2	FLEX28	567.3°	
6	32	Right	2	SYNCHRONY2	FLEX28	672.6°	Right
		Left	2.5	SYNCHRONY2	FLEX28	590.3°	
7	74	Right	3.5	SYNCHRONY2	FLEX24	693.9°	Left
		Left	15	PULSAR	STANDARD	676.1°	
8	62	Right	1	SYNCHRONY2	FLEX28	518.7°	Left
		Left	2.5	SYNCHRONY2	FLEX28	557.8°	
9	22	Right	0.5	SYNCHRONY2	STANDARD	815.5°	Left
		Left	14	PULSAR	STANDARD	792.5°	
10	34	Right	4.5	SYNCHRONY2	STANDARD	622.5°	Right
		Left	0.5	SYNCHRONY2	STANDARD	611.7°	

## Data Availability

The data presented in this study are available on request from the corresponding author.
